# Editorial: Regulators of a Regulator: Post-Transcriptional Regulation in Tregs

**DOI:** 10.3389/fimmu.2021.699911

**Published:** 2021-06-22

**Authors:** Xuyu Zhou, Li-Fan Lu

**Affiliations:** ^1^ CAS Key Laboratory of Pathogenic Microbiology and Immunology, Institute of Microbiology, Chinese Academy of Sciences (CAS), Beijing, China; ^2^ Savaid Medical School, University of Chinese Academy of Sciences, Beijing, China; ^3^ Division of Biological Sciences, University of California, San Diego, La Jolla, CA, United States

**Keywords:** post-transcriptional and post-translational regulation, Treg, non-coding RNAs, RNA modification, Foxp3

In the last 20 years, an enormous body of evidence has demonstrated critical roles of a unique subset of CD4 T cells, so-called regulatory T cells (Tregs), in maintaining immunological self-tolerance by actively suppressing aberrant immune responses. The multiple functional properties of Tregs target a variety of immune cells through a range of potent suppressive mechanisms. Moreover, it has also become clear that rather than being a homogeneous population, Tregs can undergo functional specialization in response to different environmental clues, allowing them to control local inflammation in a given tissue microenvironment. While numerous studies have established Foxp3 act as a master molecular regulator that orchestrates a transcriptional program that governs the differentiation and function of Tregs, mounting evidence has also demonstrated a crucial role of post-transcriptional mechanisms in controlling multiple aspects of Treg biology.

A series of recent contributions in *Frontiers in Immunology* have addressed post-transcriptional and post-translational mechanisms of regulating Treg development, homeostasis, activation, lineage stability, and suppressive function. In the mini-review “Effects of Non-Coding RNA on Regulatory T Cells and Implications for Treatment of Immunological Diseases”, Luo and Wang provide a comprehensive overview of individual non-coding RNAs that control Treg homeostasis and suppressive function. Non-coding RNAs, including microRNAs and long non-coding RNAs, were once considered “junk” genetic material, but extensive efforts in the last decade have revealed that key non-coding RNAs are indispensable in the generation and function of Tregs. To this end, Kunze-Schumacher and Krueger provide a broad overview of how microRNAs influence Treg development and function in their mini-review, “The Role of MicroRNAs in Development and Function of Regulatory T Cells – Lessons for a Better Understanding of MicroRNA Biology”. The network of microRNAs and their targets are extremely complex. Kunze-Schumacher and Krueger discuss miRNA-target relationships in detail, including context-dependent function and the roles of individual targets vs. complex co-targeting networks.

RNA modification, also called “RNA epigenetics” is another attractive area of research on post-transcriptional regulation. Among these modifications, RNA methylation is particularly common and abundant. With the identification of the important “writer”, “eraser” and “reader” enzymes that reversibly mediate methylation, the functions of RNA methylation in T cells, including Tregs, are now being elucidated. In their mini-review entitled “Multiple Functions of RNA Methylation in T Cells: A Review”, Chao et al. provide a detailed summary of recent progress in uncovering the effects of four major forms of RNA methylation (m6A, m6Am, m1A, and m5C) on T cell function.

Finally, two articles have provided insights on post-translational regulation in Tregs. In original research entitled “Mouse Double Minute 2 Homolog-Mediated Ubiquitination Facilitates Forkhead Box P3 Stability and Positively Modulates Human Regulatory T Cell Function”, Wang et al. demonstrated that mouse double minute 2 homolog (MDM2), an E3 ubiquitin ligase of p53, also interacts with Foxp3 and unexpectedly stabilizes Foxp3 expression. Dong et al. focus on post-translational regulation of Foxp3 in their review entitled “Post-Translational Regulations of Foxp3 in Treg cells and Their Therapeutic Applications” and provide detailed lists of Foxp3 binding patterns and post-translational modifications and their potential function in regulating Foxp3 function and plasticity.

This series of Research Topics provides an extensive update on post-transcriptional and post-translational regulation in Tregs. A deeper understanding of these molecular and cellular mechanisms that control Treg development and function would undoubtedly provide unique opportunities to treat not only various autoimmune diseases but also other immunological disorders.

## Author Contributions

XZ and L-FL contributed equally to this work. All authors contributed to the article and approved the submitted version.

## Conflict of Interest

The authors declare that the research was conducted in the absence of any commercial or financial relationships that could be construed as a potential conflict of interest.

